# Modulation of Unfolded Protein Response Restores Survival and Function of β-Cells Exposed to the Endocrine Disruptor Bisphenol A

**DOI:** 10.3390/ijms24032023

**Published:** 2023-01-19

**Authors:** Laura Maria Daian, Gabriela Tanko, Andrei Mircea Vacaru, Luiza Ghila, Simona Chera, Ana-Maria Vacaru

**Affiliations:** 1 BetaUpreg Research Group, Institute of Cellular Biology and Pathology “Nicolae Simionescu”, 050568 Bucharest, Romania; 2 Center for Diabetes Research, Department of Clinical Sciences, Faculty of Medicine, University of Bergen, 5021 Bergen, Norway

**Keywords:** unfolded protein response, diabetes, endocrine disruptor, bisphenol A, insulin granules

## Abstract

Diabetes is a metabolic disease that currently affects nearly half a billion people worldwide. β-cells dysfunction is one of the main causes of diabetes. Exposure to endocrine-disrupting chemicals is correlated with increased diabetes incidence. We hypothesized that treatment with bisphenol A (BPA) induces endoplasmic reticulum (ER) stress that activates the unfolded protein response (UPR), leading to impaired function of the β-cells, which over time, can cause diabetes. In this study, we aimed to evaluate UPR pathways activation under BPA treatment in β-cells and possible recovery of ER homeostasis. MIN6 cells (mouse insulinoma cell line) and isolated pancreatic islets from NOR (non-obese diabetes resistant) mice were treated with BPA. We analyzed the impact of BPA on β-cell viability, the architecture of the early secretory pathway, the synthesis and processing of insulin and the activation of UPR sensors and effectors. We found that the addition of the chemical chaperone TUDCA rescues the deleterious effects of BPA, resulting in improved viability, morphology and function of the β-cells. In conclusion, we propose that modulators of UPR can be used as therapeutic interventions targeted towards regaining β-cells homeostasis.

## 1. Introduction

Diabetes is characterized by β-cell failure to meet insulin demand due to either β-cell dysfunction or loss of β-cell mass, which leads to ineffective insulin secretion (for type 1 diabetes—T1D), or by the development of insulin resistance due to abundant insulin secretion, but which for various reasons cannot be properly used by the body (T2D). Risk factors that lead to the appearance of diabetes can be genetic factors, but also a sedentary lifestyle or an unhealthy diet [1,2,3]. In recent decades, diabetes has become a disease that affects more and more people, and only the premise of an unhealthy lifestyle can no longer support this increase in incidence. Endocrine-disrupting compounds in the environment are hypothesized to play a role in the continued increase in metabolic disease. These are chemical compounds synthesized by humans, which once they reach the environmental compartments, can bioaccumulate and have toxic effects on living things and humans.

Bisphenol A (BPA) is a synthetic compound used in the manufacturing of polymers that are found in bottles, electronics, sports equipment, and in epoxy resins coating the interior of food cans [4] or as a constituent of thermal paper used for cash register receipts [5]. Human exposure to BPA is ubiquitous and can occur through ingestion of contaminated foods or beverages or by dermal absorption [6]. BPA can migrate from plastics or epoxy resins in foodstuffs when maintained at high temperatures [7]. Once in the body, BPA presents a short biological half-life, being absorbed in the gastrointestinal tract and conjugated in the liver to BPA-glucuronide or BPA-sulphate, polar compounds that are excreted through the urinary tract [8]. However, this compound can be deconjugated by specific enzymes leading to circulating “free” BPA [9,10] that enters the cell through a multitude of receptors [11]. Epidemiological studies revealed that in 90% of urine samples from individuals, there were traces of BPA, indicating a basal contamination with this compound [12]. BPA is associated with metabolic disorders [13,14,15,16], with a wide range of toxic effects in the body, including the disturbance of cell homeostasis by oxidative stress injury [15,17], increasing autophagy [18], mitochondrial damage [16] or endoplasmic reticulum (ER) stress.

ER is the organelle where correct folding and processing of proteins occurs through chaperons and enzymes involved in post-translational modifications. However, in conditions that disrupt metabolic homeostasis, there is an increase in misfolded proteins leading to ER stress. The UPR is the ER reaction to an accumulative stress. UPR is a well-conserved molecular mechanism comprising of three ER sensors—protein kinase RNA (PKR)–like ER kinase (Perk), inositol requiring enzyme 1 (Ire1) and activating transcription factor 6 (Atf6)—activated by the build-up of misfolded proteins [19]. The three transmembrane proteins are sensitive to Bip/GRP78, a chaperon that tags the defective proteins and dissociates from the UPR sensors, where usually it is found in ER homeostasis conditions. Perk phosphorylates the eukaryotic translation initiation factor 2α (eIF2α), thus globally inhibiting translation, with the exception of Atf4, which is induced. Atf4 upregulates the transcription of different chaperons or redox enzymes for the restoration of ER homeostasis—the adaptive UPR. When the ER stress conditions are prolonged, Atf4 enhances the synthesis of transcription factor C/EBP homologous protein (Chop), which leads the cellular fate to apoptosis, hence the apoptotic UPR. Ire1 is a protein with dual function, as a kinase and RNase. Upon activation, Ire1 trims an intron from X-box binding protein 1 (Xbp1) mRNA. The resulting protein, Xbp1s, acts as a transcription factor for various chaperons, proteins involved in the lipid biosynthesis and different components from the ER-associated degradation (ERAD) complex leading to a decrease in misfolded protein load in the ER. The third sensor, Atf6, after activation, is translocated in the Golgi apparatus and cleaved by proteases (site-1 and site-2 proteases), resulting in the N-terminal cytosolic fragment, Atf6 (N), a transcription factor that upregulates Bip, protein disulfide isomerase and other chaperons [20,21,22].

Proinsulin is produced in the rough ER lumen from pre-proinsulin, travels through the Golgi apparatus and matures into active insulin with the help of cellular endopeptidases that cleave a fragment called C-peptide, and other two peptide chains, B- and A- that oligomerize. All these processes take place in distinct regions of the trans-Golgi apparatus called insulin granules [23,24,25]. The latter are considered immature as long as they contain mostly unprocessed proinsulin. Accumulations of immature granules have been associated with diabetes [23,26].

Routinely, β-cells manage up to 20% of newly synthesized misfolded proinsulin, which represents 30–50% of the total translational load of the cell, mostly due to a well-adapted UPR [27]. However, when additional stresses occur in the ER, the UPR cannot re-establish homeostasis, and thus the ER stress can determine β-cell failure leading to diabetes [28]. One of the hallmarks of ER stress observed at the ultrastructural level, also in β-cells, is the dilated ER, correlated with an expanded Golgi apparatus [23,26].

Several studies provided information that BPA activates an ER stress in different types of cells, such as mouse non-parenchymal hepatocytes [29] and human endometrial stromal cells [30]. In addition, studies on non-obese diabetic (NOD) mice correlate BPA and its derivatives exposure with an increased incidence of diabetes [31,32,33]. The hypothesis of this study was that BPA would affect the insulin-secreting β-cells by inducing an ER stress response, which could not be ameliorated by an adaptive UPR, leading to a dysfunctional cell with severe consequences in diabetes. We also aimed to counter the negative effects of BPA by utilizing a UPR modulator, tauroursodeoxycholic acid (TUDCA). By using a mouse insulinoma cell line (MIN6) and isolated islets from non-obese resistant (NOR) mice, we show that BPA significantly affects β-cell viability and function through a pro-apoptotic UPR and that TUDCA is a good candidate to maintain β-cell homeostasis under stressed conditions.

## 2. Results

### 2.1. BPA Exposure Induces Death of Both MIN6 Cells and Isolated Mouse Pancreatic Islets

**Figure 1:**
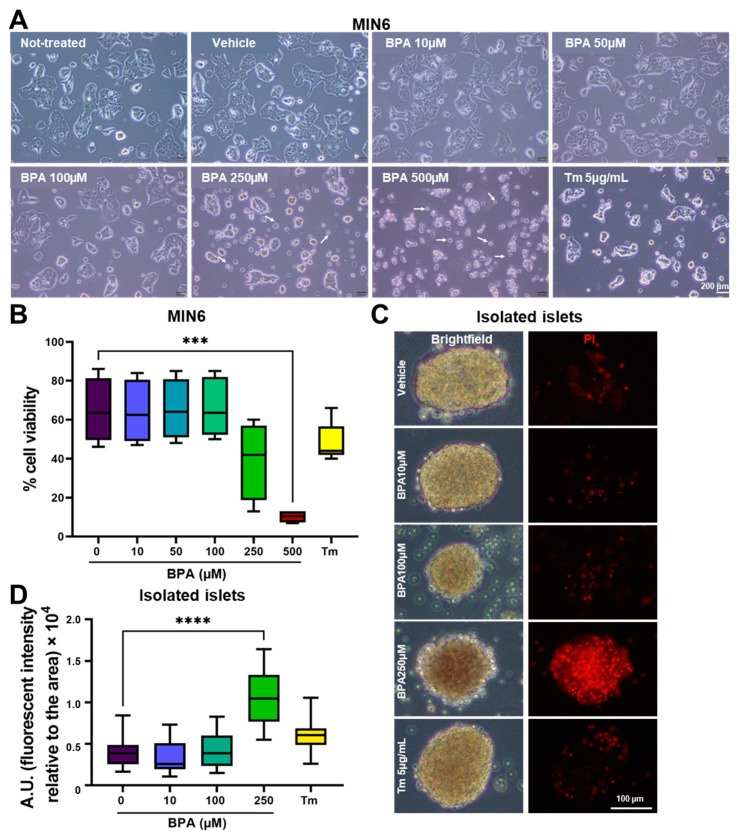
Treatment with increasing concentrations of BPA reduced MIN6 and islet cell viability. (**A**) Brightfield microscopy images of MIN6 cells after 24 h of treatment with increasing concentrations of BPA (0–500 µM) and with 5 µg/mL Tm, respectively. Apoptotic/dead cells are indicated by arrows. The scale bar is 200 μm. (**B**) Graph depicting cell viability as determined by flow cytometry analysis upon staining with Annexin V and PI for MIN6 cells treated with BPA (0–500 µM) or with 5 µg/mL Tm. (**C**) Fresh islets isolated from NOR mice were treated for 24 h with increasing concentrations of BPA (0–250 µM) or 5 µg/mL Tm and stained with PI. Representative brightfield microscopy images (left) and fluorescence microscopy images of the islets labeled with PI (right) after 24 h of various treatments as described above. The scale bar is 100 µm. (**D**) Graph showing analysis of cell death in the islets (*n* = 21 per condition) expressed as a ratio of PI fluorescence intensity and islets aria, the y axis represents A.U. *** *p* < 0.0005, **** *p* < 0.0001 by One-way ANOVA.

To study the effect of BPA on β-cells’ viability, we have treated MIN6 cells with increasing concentrations of BPA. Untreated and vehicle (ethanol)-treated cells grow in small clusters that tend to fuse to enlarged aggregates, without reaching full confluency, with very few cells that start to detach from the cluster and die (Figure 1A, not-treated and vehicle). Upon treatment with BPA, it becomes apparent that cells cluster less while more single cells, as well as round cells, marked by arrows, are present. This is similar to the cells exposed to the ER stress inducer Tunicamycin (Tm), which served as a reference for stress induction (Figure 1A, 100–500 µM BPA and Tm 5 µg/mL).

Cells exposed for 24 h to stressors were labeled with Annexin V and propidium iodide (PI) to mark the apoptotic and the dead cells, respectively, then analyzed by flow cytometry. Viable cells were considered all the cells gated as in Figure S1A,B, which were negative for both Annexin V and PI (lower left quadrant from the dot plots presented in Figure S1B). The percentages of viable cells exposed to concentrations of BPA ranging between 10 and 100 µM were not affected significantly, as it varied around 65%, being comparable with that of the vehicle control-treated cells (Figure 1B and Figure S1B,C). Treatment with 250 and 500 µM BPA determined a decrease in percentages of viable cells from 65% (vehicle control) to ~40% and 10%, respectively. This is due to the induction of apoptosis, as we found an increase in Annexin V-positive cells from ~20% for the vehicle-treated cells to 50% and 70%, respectively (Figure 1B and Figure S1B,C). A similar decrease in viability was observed when MIN6 cells were treated with a known ER stressor, Tm, which reduced the percentage of viable cells to around 45% (Figure 1B and Figure S1B,C).

Next, we analyzed if the BPA effects are similar on the viability of cells from freshly isolated islets of Langerhans from NOR mice. After 24 h of treatment with up to 250 µM BPA and with 5 µg/mL Tm, respectively, islets were stained with PI and quantified. Representative images of islets are presented in Figure 1C. Culturing freshly isolated islets for 24 h does not induce a significant number of dead cells (Figure 1C,D, vehicle). Similar results were obtained after treatment with BPA 10 and 100 µM. However, upon treatment for 24 h with 250 µM BPA, the islets showed a significant accumulation of approximately three-fold more PI-labeled cells than in vehicle-treated cells, which was quantified and expressed as the fluorescence intensity of PI relative to the islet surface (Figure 1D). Similarly, we found that treatment with Tm significantly decreased islet cell viability by almost twofold as compared to the vehicle-treated cells (Figure 1C,D, Tm 5 µg/mL).

### 2.2. The Morphology of the Early Secretory Pathway in MIN6 Cells Is Affected by BPA

Most extracellular proteins are processed through the secretory pathway to becoming biologically active. As a secreted hormone, insulin follows this pathway being produced by the β-cells in the ER and transported to the Golgi apparatus, where it is further processed and packaged in the secretory granules (SG). After glucose stimulation, SG merges with the plasma membrane to release insulin into circulation [34]. As the ER and the Golgi apparatus are important steps in insulin processing, we aimed to analyze the effect of BPA exposure on the early secretory pathway of MIN6 cells. The ER and the Golgi apparatus were identified by immunostaining against calnexin, a membrane protein in the ER, and against GM130, a structural component of the cis-Golgi apparatus, respectively.

In the cells exposed to the vehicle, calnexin displays a network pattern accompanied by puncta and localized accumulations, which are characteristic of this marker. The Golgi apparatus in control cells showed a specific appearance, a ribbon-like, compact structure located on one side of the cell. Upon 24 h of treatment with concentrations of BPA higher than 100 µM, alterations in both these structures were observed. As such, the fluorescence intensity of calnexin is decreased at 100 µM BPA and becomes diffuse at 250 µM BPA, indicating the disassembly of the ER network. Similarly, treatment with 100 µM BPA produced a scattered pattern for the GM130 staining, while at 250 µM BPA, the GM130 labeling was almost lost, suggesting the disintegration of the Golgi complex (Figure S2). All these perturbations may lead to potential problems in protein processing.

**Figure 2:**
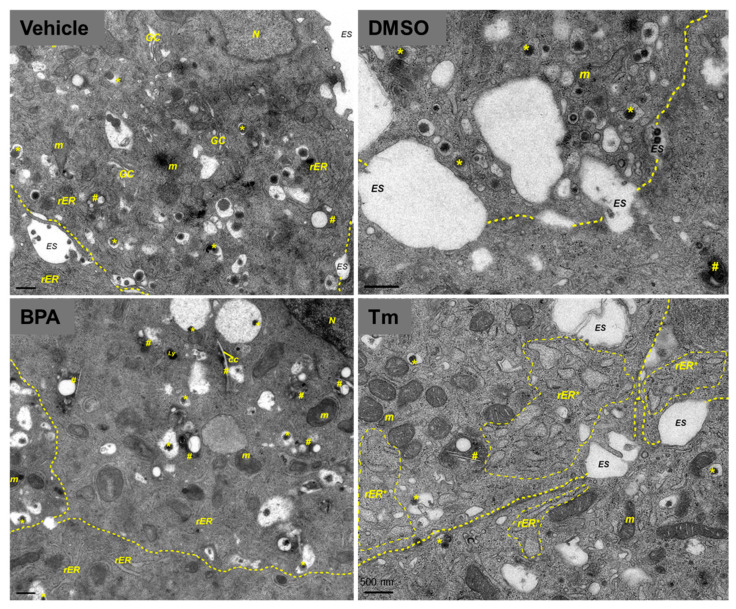
BPA exposure induces ultrastructural changes in MIN6 β-cells. Transmission electron microscopy images of MIN6 cells cultured for 24 h in the presence of vehicle for BPA (vehicle (ethanol), top left), 100 µM BPA (bottom left), a vehicle for Tm (DMSO, top right), and 5 µg/mL Tm (bottom right). Yellow dotted lines show boundaries between adjacent cells, while lighter yellow dashed lines indicate distended rough endoplasmic reticulum (rER). Insulin granules (*), autophagosomes (#). Note the abundant lipid-like droplets inside autolysosomes and the occurrence of cholesterol crystal (cc) clefts in BPA-exposed cells, suggestive of disturbed lipid metabolism after BPA exposure. The scale bar is 500 nm. *N*, nucleus; *m*, mitochondria; *Ly*, lysosomes; *ES*, extracellular space.

To investigate in more detail how MIN6 cells are affected by BPA at the ultrastructural level, we performed conventional electron microscopy (EM). MIN6 cells treated with the control vehicles displayed a normal β-cell morphology. The insulin secretory granule showed a distinctive appearance, where the electron-dense crystalline core was surrounded by an electron-lucent halo and enclosing membrane ([35]; Figure 2 vehicle, DMSO). In contrast, the majority of insulin granules from the 100 µM BPA-treated cells displayed an altered structure, with eccentric electron-dense cores, wide granule halos with proteinaceous appearance, and/or fuzzy borders (Figure 2, BPA). Furthermore, in the cytoplasm of MIN6 cells exposed to BPA, we noticed slightly enlarged ER tubules, and numerous autophagosomal structures (autophagosomes and autolysosomes) containing lipid-like droplets, and sometimes cholesterol crystal clefts. We used treatment with 5 µg/mL Tm as a reference for the detection of morphological defects produced by ER stress. The EM images of MIN6 cells exposed to Tm revealed a diminished population of insulin secretory granules, having altered morphology and other dramatic changes, such as cisternal distension of the ER and the Golgi apparatus, as well as swelling of mitochondria (Figure 2, Tm).

### 2.3. BPA Leads to Impaired Insulin Synthesis

**Figure 3:**
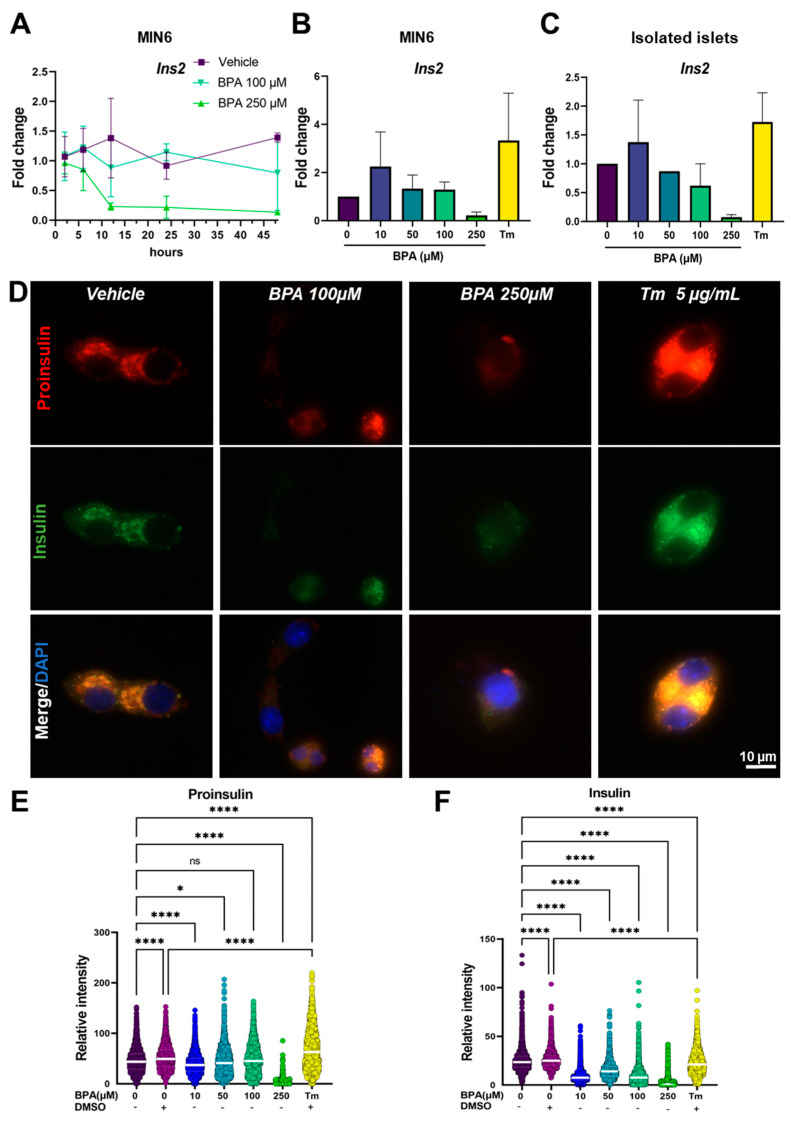
Insulin processing is affected by BPA and Tm in MIN6 cells and isolated islets. (**A**) Time course of BPA-induced *Ins2* gene expression at different time points (2, 6, 12, 24, 48 h) in MIN6 cells. RT-qPCR analysis of the *Ins2* gene in MIN6 cells (**B**) and isolated islets (**C**) after 24 h of treatment with different concentrations of BPA and 5 μg/mL Tm. Fold change values were calculated by normalization to *Gapdh* and then to the NT values. (**D**) Representative fluorescence microscopy images with MIN6 cells after 24 h of treatment with 100, 250 μM BPA and 5 μg/mL Tm, marked for proinsulin (red) and insulin (green). The nucleus was stained with DAPI. (**E**) Graphic representation of the mean intensity of the red fluorescent channel (proinsulin) and (**F**) the mean intensity of the green fluorescent channel (insulin) per cell (*n* > 200 cells) in MIN6 cells after 24 h of treatment with increasing concentrations of BPA (as shown) and 5 μg/mL Tm. The mean intensity was determined with QuPath. Graphs display the median and the 25th and 75th percentiles. * *p* < 0.05, **** *p* < 0.0001, ns—not significant, based on one way ANOVA.

As morphology is tightly correlated with function, especially in highly secretory cells such as the β-cells, we set out to analyze what is the impact of the disturbances caused by BPA on insulin production. To determine the dynamics of insulin synthesis in MIN6 cells exposed to 100 and 250 µM BPA, we performed a time course experiment following the predominant mouse insulin gene, *Ins2*, mRNA expression at different time points (2, 6, 12, 24 and 48 h) (Figure 3A). We found two peaks of *Ins2* gene synthesis at 12 and 48 h, respectively, for both untreated cells and cells exposed to the vehicle. Interestingly, in cells incubated with 100 µM BPA, *Ins2* does not display any significant variation during the 48-h treatment. However, 250 µM BPA treatment determined a decrease in insulin mRNA synthesis, observed after 6 h of exposure by a threefold decrease in gene expression compared to the control. After 12 h of treatment, it reached a tenfold decrease in gene expression and remained constant until the end of the experiment (48 h). In addition, for the 24 h time point, we analyzed *Ins2* gene expression both in MIN6 cells and in isolated islets upon exposure to concentrations of BPA ranging between 10 and 250 µM and 5 µg/mL Tm, respectively, used as an ER stress control (Figure 3B,C). In both biological systems, we found a similar trend, where the lowest concentration of BPA used (10 µM) modestly increased the expression of the gene by approximately twofold, while a high concentration (250 µM) inhibited the synthesis with a decrease of 14-fold compared to control. Additionally, we found that treatment with Tm produced a fourfold increase in *Ins2* gene expression in MIN6 cells and a twofold increase in isolated islets, most probably due to a positive feedback loop (Figure 3B,C, Tm).

Next, to analyze how BPA affects the capacity of MIN6 cells to process insulin after 24 h of treatment, we immunostained the cells with antibodies targeting proinsulin (red) and insulin (green) (Figure 3D). For cells treated with the vehicle, we observed a tight correlation between the fluorescence intensity of proinsulin to the intensity of insulin (Figure 3D–F, Vehicle). Moreover, for cells treated with concentrations of BPA ranging between 10 and 100 µM, proinsulin synthesis did not show large variations (from 45.9 ± 22.9, *n* = 2359 cells (vehicle) to 39.0 ± 19.7, *n* = 4743 cells (BPA 10 µM); to 43.8 ± 26.5, *n* = 2353 cells (BPA 50 µM); to 47.8 ± 28.5, *n* = 2575 cells (BPA 100 µM) (Figure 3D,E). However, we found that treatment with 250 µM BPA leads to a significant decrease in the fluorescence intensity of proinsulin, indicating an impairment in the synthesis of the hormone (Figure 3D, BPA 250 µM), the result also supported by the quantification of proinsulin fluorescence intensity from 45.9 ± 22.9, *n* = 2359 cells (vehicle) to 3.3 ± 7.7, *n* = 692 cells (Figure 3E). The exposure to 5 µg/mL Tm resulted in an increased synthesis and accumulation of proinsulin, from 51.9 ± 22.9, *n* = 2277 cells (DMSO) to 68.4 ± 40.0, *n* = 1457 cells (Figure 3D,E, Tm 5 µg/mL). One explanation can be the fact that Tm inhibits the normal processing of proteins by disrupting the N-glycosylation process, thus producing a backlog of proinsulin in the ER. Additionally, our results indicated that further insulin processing was disrupted in the MIN6 cells starting with the lowest BPA concentrations (10 µM), as we registered a decrease in insulin fluorescence intensity from 25 ± 10.9, *n* = 2359 cells (vehicle) to 8.7 ± 5.9, *n* = 4743 cells (Figure 3F). The most affected disruption of the normal processing of this hormone was seen for MIN6 cells exposed to 250 µM BPA, where both proinsulin and insulin (from 25 ± 10.9, *n* = 2359 cells (vehicle) to 3.8 ± 7.0, *n* = 692 cells) expressions were almost completely blocked by the drug (Figure 3D–F).

### 2.4. BPA-Exposed Cells Display A Pro-Apoptotic UPR

**Figure 4:**
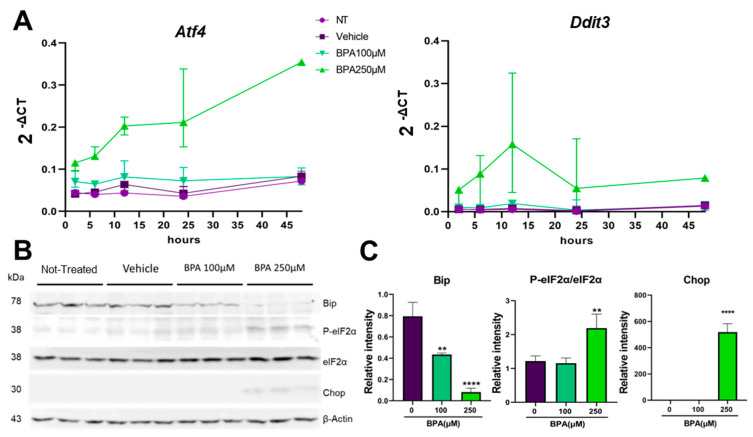
BPA determines the increased expression of pro-apoptotic UPR elements. (**A**) Time course of BPA-induced gene expression of *Atf4* and *Ddit3* in MIN6 cells. The 2^−ΔCT^ values were calculated by normalization to *Gapdh*. (**B**) MIN6 cells were treated for 8 h with BPA (100 or 250 µM). Whole-cell lysates were analyzed by Western blot for GRP78 (Bip), P-eIF2α, total eIF2α, Chop and β-Actin. (**C**) Graphs depicting quantifications of the immunoblots where the values were first normalized to β-Actin and then to the values of the untreated group. ** *p* < 0.005, **** *p* < 0.0001 compared to the vehicle by One-way ANOVA. Graphs represent the mean ± SD.

We next asked what is the mechanism that BPA employs to affect the function of MIN6 cells. One potential clue was given from the disturbances of the secretory pathway that we observed in the cells exposed to BPA. As the early secretory pathway was perturbed, we analyzed the stress response from the ER at the molecular level, named the unfolded protein response, or the UPR. Thus, we examined the three main UPR branches by following the sensors, Atf6, Ire1 and Perk, or their downstream effectors. For that, we evaluated the cells during 48 h of treatment with 100 and 250 µM BPA, respectively, and analyzed them at various time points, as previously described. The results presented in Figure S3 show that upon treatment with BPA, two of the UPR signaling pathways were not significantly upregulated by the compound after 24 h. Both Atf6 and Ire1 branches evaluated by RT-qPCR showed no upregulation of the *Atf6* gene expression and its effectors *Edem1* and *Dnajc3*, as well as Ire1 downstream targets *uXbp1* and *sXbp1* (Figure S3). However, we found the third UPR branch, the Perk pathway, upregulated upon exposure to BPA. Figure 4A shows the activation of the genes coding for Perk effectors, *Atf4* (Atf4) and *Ddit3* (Chop), upon exposure to 100 and 250 µM BPA, respectively. BPA 100 µM induces a similar trend of gene expression as the controls (not treated (NT) and vehicle) for both *Atf4* and *Ddit3* genes. On the other hand, treatment with 250 µM BPA strongly increased the expression of these two genes. At 2 h after exposure, we observed a twofold increase in *Atf4* and a sevenfold increase in *Ddit3* compared to the control. After 12 h of treatment, *Atf4* was increased from twofold to fourfold, while *Ddit3* increased from sevenfold to 28-fold, reaching the peak expression of this gene in these conditions. From this point on, *Atf4* expression remained constant at 24 h and increased from fourfold to almost eightfold at 48 h, compared to the control. Meanwhile, *Ddit3* expression decreased at 24 h from 28-fold to 18-fold and remained constant onwards (Figure 4A,B).

Additionally, we monitored the activity of Atf4 by using a sensor for the pathway that was expressed into the cells upon transfection with pLVX-ATF4 mScarlet NLS plasmid. This sensor leads to the expression of a mScarlet-I fusion protein in the nucleus upon ER-stress stimulation. The activation of the ER-stress-sensitive PERK/ATF4 pathway can be monitored by recording the red fluorescent signal arising in the nucleus. Transfected MIN6 cells were incubated with different concentrations of BPA for 6 h and visualized for the fluorescent activation of Atf4 (Figure S4). The transcription factor activity was evaluated by the ratio between the fluorescence intensity to the area of each cell cluster. This assay showed an increase in Atf4 activity in the 250 µM BPA-treated cells, from 2.741 × 10^−4^ ± 3.6 × 10^−4^, *n* = 66 cell clusters, to 4.642 × 10^−4^ ± 4.2 × 10^−4^, *n* = 103 cell clusters; however, not significant (Figure S4A,B). Moreover, when we used Tm as an ER stress-inducer, we found that it was significantly upregulated, from 2.741 × 10^−4^ ± 3.6 × 10^−4^, *n* = 66 cell clusters to 5.018 × 10^−4^ ± 5.6 × 10^−4^, *n* = 102 cell clusters, confirming the efficiency of the sensor. These data were confirmed by Western blot analysis. As we previously showed that Perk downstream gene targets were upregulated starting with 2 h of exposure to BPA, we evaluated protein expression of Bip, phosphorylated-eIF2α (P-eIF2α), total eIF2α and Chop after 8 h BPA treatment (Figure 4B,C). We found that Bip was significantly decreased by twofold in cells treated with 100 µM BPA, and approximately eightfold lower in cells treated with 250 µM BPA, respectively, as compared to the vehicle-treated cells. Although we found that the ratio between P-eIF2α to total eIF2α did not change in cells exposed to 100 µM BPA in cells exposed to 250 µM BPA, it significantly increased by twofold as compared to the control. Similarly, we found Chop expression was significantly induced after 8 h of exposure to the 250 µM BPA, as compared to the vehicle-treated cells (Figure 4B,C).

### 2.5. TUDCA Improves Viability of BPA-Compromised MIN6 Cells

**Figure 5:**
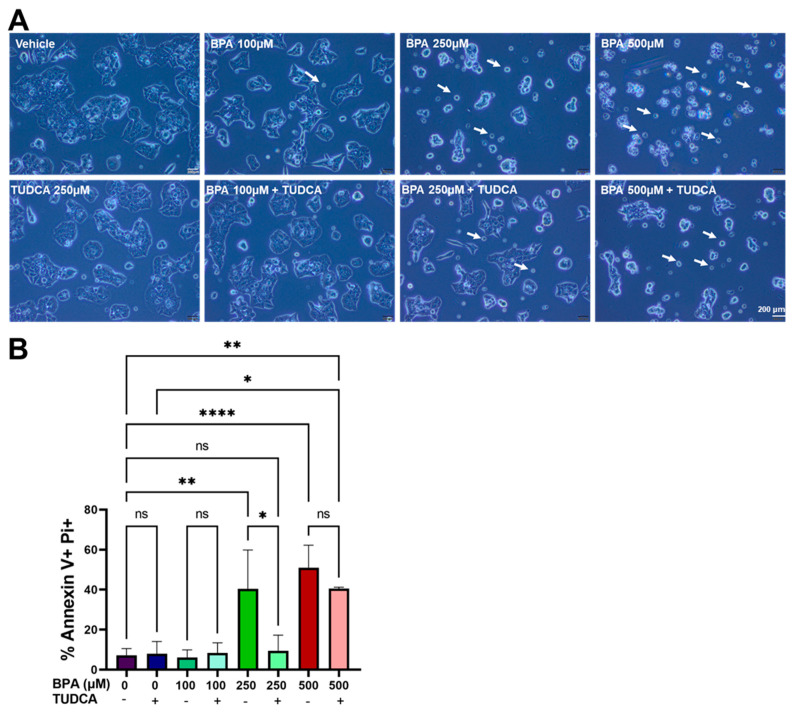
Co-treatment of BPA with TUDCA reduces the death of MIN6 cells. (**A**) Representative brightfield microscopy images with MIN6 cells after 24 h of treatment with BPA (0–500 µM) in the presence or absence of TUDCA 250 µM. (**B**) Apoptosis of MIN6 cells exposed to BPA and TUDCA was determined by flow cytometry upon co-staining with Annexin V and PI. ns—not significant, * *p* < 0.05, ** *p* < 0.005, **** *p* < 0.0001 based on One way ANOVA. Graphs represent the mean ± SD.

Previous studies showed that bile acid TUDCA increased β-cell survival [36,37]. To evaluate the beneficial effect of TUDCA, we employed this molecule in MIN6 cells exposed to increasing concentrations of BPA (100, 250 and 500 µM) and determined their viability. First, we found that cells treated with 250 µM TUDCA were similar to the control cells (Figure 5A, vehicle, TUDCA 250 µM). When combined with BPA, we found that TUDCA diminished the occurrence of apoptotic cells (marked by white arrows) and partially prevented the morphological changes of the clustered cells (Figure 5A). Flow cytometry analysis showed that co-treatment of BPA with TUDCA significantly decreased the percentage of cells double-positive for PI and Annexin V, from 40% in the cells treated with 250 µM BPA to approximately 10% in the cells co-treated with 250 µM BPA and 250 µM TUDCA, respectively (Figure 5B and Figure S4).

### 2.6. Co-Treatment of MIN6 Cells with BPA and TUDCA Regulates Perk Downstream Effectors

We hypothesized that TUDCAs mechanism of action for maintaining cell viability in the presence of BPA is by improving protein folding in the ER and thus inhibiting the activation of a pro-apoptotic UPR response.

**Figure 6:**
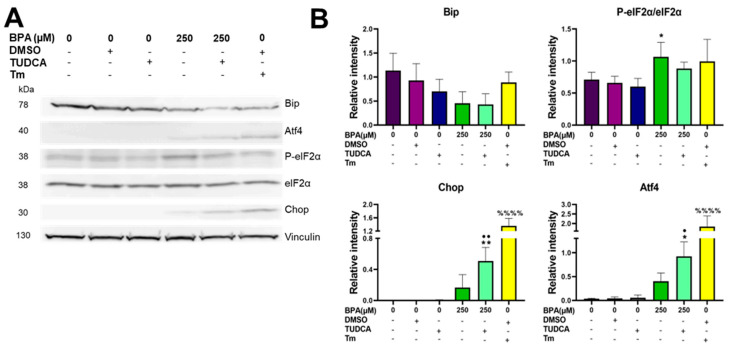
The UPR branch Perk is activated when co-treating cells with 250 µM BPA and 250 µM TUDCA. (**A**) MIN6 cells were treated for 8 h with BPA, TUDCA, DMSO and 5 µg/m Tm. Whole-cell lysates were analyzed by Western blot for Bip, Atf4, P-eIF2α, total eIF2α, Chop and Vinculin. (**B**) Graphs depict quantifications of the immunoblots. Vinculin was used as a normalizer. * *p* < 0.05, ** *p* < 0.005, compared to vehicle; ^%%%%^ *p* < 0.0001 compared to DMSO; • *p* < 0.05, •• *p* < 0.005 compared to TUDCA, based on One way ANOVA. Graphs represent mean ± SD.

We analyzed the UPR targets in cells acutely exposed, for 8 h, to both BPA and TUDCA. We found that the addition of TUDCA did not rescue Bip downregulation observed in 250 µM BPA-treated cells (Figure 6A,B, Bip). Interestingly, the ratio between P-eIF2α and eIF2α was significantly increased by two-fold in the MIN6 cells exposed to 250 µM BPA and was restored to normal upon TUDCA addition (Figure 6A,B, P-eIF2α/eIF2α). Concomitantly a two-fold increase was observed for Atf4 as well as a three-fold increase for Chop following TUDCA addition as compared to 250 µM BPA treatment alone. These results were mirrored by the RT-qPCR analysis, where we found both *Atf4* and *Ddit3* genes increased in TUDCA-supplemented BPA-treated MIN6 cells, as compared to the BPA-treated only, albeit not significantly (Figure S6).

Additionally, we observed that the upregulation of the pro-apoptotic factors Atf4 and Chop in MIN6 cells co-treated with BPA and TUDCA did not significantly influence the expression of the downstream targets such as *Bcl2*, *Bax* and *Bad* (Figure S6).

These results indicate that TUDCA activates the Perk signaling pathway, but in a different way than BPA, ameliorating the pro-apoptotic effects the endocrine disruptor has on MIN6 cells.

### 2.7. Ultrastructure and Function of BPA-Exposed MIN6 Cells Are Improved by Addition of TUDCA

**Figure 7:**
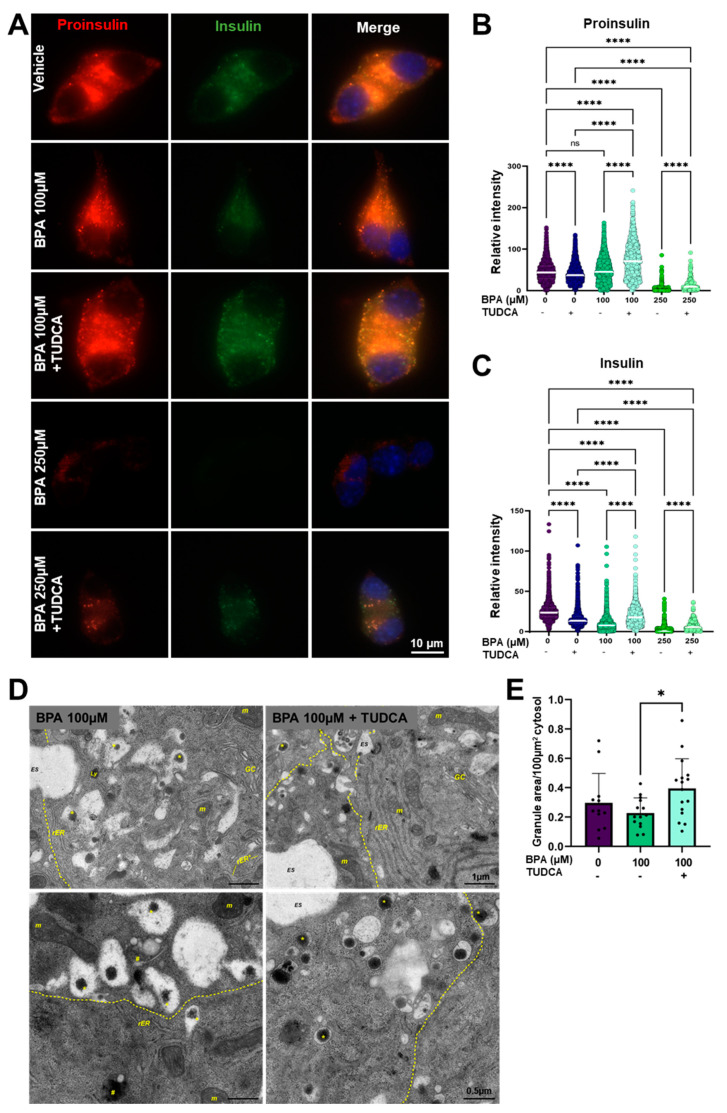
TUDCA improves insulin synthesis and processing in MIN6 cells treated with BPA. (**A**) Representative fluorescence microscopy images with MIN6 cells after 24 h of treatment with 100 and 250 μM BPA, respectively, in the presence or absence of 250 μM TUDCA, marked for proinsulin (red) and insulin (green). The nucleus was stained with DAPI. The scale bar is 10 μm. Graphic representation for the mean intensity of the red fluorescence channel (proinsulin) (**B**) and of the green fluorescence channel (insulin) (**C**) quantified per cell after 24 h of treatment of MIN6 cells with 100 or 250 μM BPA in the presence, or absence of TUDCA. Relative intensity was determined with QuPath. Graphs display the median and the 25th and 75th percentiles. ns—not significant, **** *p* < 0.0001, by One-way ANOVA. (**D**) Representative EM images of MIN6 cells treated with BPA (left) and with BPA and TUDCA (right), for 24 h. Scale bars are top: 1 µm; bottom: 500 nm. Insulin granules (*), cisternal distension of the rough endoplasmic reticulum (*rER* *); Golgi complex (*GC*); mitochondria (*m*); lysosome (*Ly*); autophagosomal structure (#); extracellular space (*ES*). (**E**) Quantification of the ratio between the surface of the insulin granule to 100 μm^2^ cytosol in the MIN6 cells treated as above. At least 12 different EM images were quantified per each condition. * *p* < 0.05 by One-way ANOVA. Graphs represent mean ± SD.

We next asked whether TUDCA treatment is beneficial for the architecture in correlation with the function of MIN6 cells. For that, we performed the analysis of proinsulin (red) and insulin (green) fluorescence intensities in the cells treated with vehicle and BPA in the presence or absence of TUDCA. The results revealed that the presence of TUDCA in cells treated with BPA significantly improved the expression levels of both proinsulin (from 47.8 ± 28.5, *n* = 2575 cells (BPA 100 µM) to 72.9 ± 41.1, *n* = 1275 cells (BPA 100 µM + TUDCA), and from 3.2 ± 7.7, *n* = 692 cells (BPA 250 µM) to 13.8 ± 14.4, *n* = 210 cells (BPA 250 µM + TUDCA) (Figure 7A,B), and insulin (from 9.2 ± 7.9, *n* = 2575 cells (BPA 100 µM) to 19.5 ± 11.9, *n* = 1275 cells (BPA 100 µM + TUDCA) and from 3.8 ± 7, *n* = 692 cells (BPA 250 µM) to 7.7 ± 6.4, *n* = 210 cells (BPA 250 µM + TUDCA) (Figure 7A,C). We next investigated whether co-treatment of TUDCA with BPA overcomes the morphological alterations noticed in BPA-exposed MIN6 cells. In Figure 7D, MIN6 cells co-treated with BPA and TUDCA displayed a rather normal ultrastructural appearance, with an abundant content of mature insulin secretory granules presenting a typical morphology and a regular rough ER. Quantification of the total area of secretory granules per given cytosolic area revealed significant differences between BPA alone and BPA plus TUDCA-treated cells, while no differences were evidenced between control cells and cells co-treated with BPA and TUDCA (Figure 7E). Taken together, these results demonstrate the rescue potential of TUDCA on BPA-exposed MIN6 cells.

## 3. Discussion

Previous studies showed that pancreatic β-cells can be a target for the toxic effects of BPA [38,39,40]. However, some of these studies focus on BPAs property to act as a weak agonist for estrogen receptors. In recent years it was noted that BPA can interact with a wide variety of receptors [11]. Thus, BPA-induced dysfunction, which can result in apoptosis of the pancreatic β-cells, might not happen through one specific mechanism but rather through the concerted action of multiple ones that still need thorough characterization.

In this study, we hypothesized that BPA interferes with insulin processing in the ER and thus activates a pro-apoptotic UPR. By stimulating the cells with a chemical that acts as a chaperone, TUDCA, we intended to improve the fault in protein processing and restore insulin homeostasis. To test this hypothesis, we exposed MIN6 cells and isolated pancreatic islets to different concentrations of BPA in the absence or presence of TUDCA and evaluated β-cells viability, UPR activation, morphology and function. Our study showed that treatment with a high concentration of BPA resulted in increased apoptosis through the activation of the pro-apoptotic UPR, as well as impaired insulin synthesis and processing. Co-treatment with TUDCA improved the cell function and viability.

We evaluated the cytotoxicity of BPA on two different models, MIN6 cells and freshly isolated mouse pancreatic islets, by flow cytometry upon staining MIN6 cells with Annexin V and PI (Figure 1A,B and  Figure S1) and by fluorescence microscopy when assessing the PI-positive cells from the islets, respectively (Figure 1C,D). Regardless of the system we used, we found appreciable cell death at 250 µM BPA and higher. Meanwhile, low doses of BPA (10–100 µM) had no significant effect on viability. The concentrations used in our experiments are higher than the estimated daily intake of 0.01 to >5 µg/kg BW/day for adults [41,42,43]. Nonetheless, BPA exposure is ubiquitous. After entering the body, BPA undergoes biotransformation to BPA-glucuronide by conjugation with glucuronic acid or BPA-sulphate as Phase II metabolites. These compounds are very polar, facilitating their excretion in urine [44]. However, there have been reported cases when enzymatic deconjugation resulted in circulating “free” BPA [10,45]. Considering the wide range of products associated with BPA, we may assume that human exposure is constant. Thus, it is worth testing concentrations greater than the ones labeled safe. The concentrations chosen for this study are comparable with doses that induce apoptosis in other studies on similar cell types (INS-1) [38] or different cell types (NCTC Clone 1469—mouse non-parenchymal hepatocytes, IMR-32 and SK-N-SH—human neuroblastoma cell lines) [29,46]. It should be mentioned that in a recent study, Al-Abdulla and collaborators using MIN6 cells grown in similar conditions and under a similar treatment time with BPA (24 h) observed a decreased cell viability at concentrations as low as 10 and 100 nM BPA [47]. There are also studies that noticed a variety of effects, including apoptosis induced by BPA at concentrations in the nM range in isolated rat islets [48]. One possible explanation for this variety of results may come from the non-monotonic dose response that this endocrine disruptor displays [49].

Ultrastructural analysis of MIN6 exposed to BPA could provide some insights into how BPA exposure disturbs the insulin secretory pathway and produces β-cell dysfunction. Previous reports suggested that BPA exposure could modify the expression of key proteins involved in the ER stress response [50] and induce morphological changes of the ER, affecting the pathway from protein assembly to secretion [29]. Considering that the accumulation of unfolded/misfolded proteins in ER lumen in the process of ER stress increases the demand for autophagic removal and activates autophagy [51], it is conceivable to assume a causal link between BPA and the build-up of large autophagosomal structures noticed in BPA-exposed MIN6 cells (Figure 2, BPA). Noteworthy, the density volume of the autophagic structures is increased either by enhanced autophagic activity or restrained autophagic flux at the lysosomal step [52]. Mounting evidence has shown that exposure to BPA impairs lysosomal acidification and suppresses autophagic flux [53,54]. The pH of insulin granules is acidic, and proinsulin conversion to insulin requires an acidic compartment [55]. So, it can be inferred that BPA exposure may hinder secretory vesicles to become more acidic and intervene in the processing of proinsulin and storage of insulin within the dense core granules in the pancreatic β-cell. Our results support this proposed mechanism, as we found defects in the early secretory pathway both by immunofluorescence and by EM. MIN6 cells exposed to BPA displayed diffuse and scattered staining of calnexin (ER marker) and of GM130 (Golgi apparatus marker), respectively (Figure S2). Moreover, by EM, we found that, besides enlarged ER tubules, BPA alters the structure of the insulin granules, conferring them an eccentric electron-dense core, wide granule halos with a protein crystals appearance and with indistinct margins (Figure 2, BPA). Additionally, we confirmed that Tm, a *bona fide* ER stress inducer, produced a dramatic effect on the ER and the Golgi apparatus, inducing well-documented cisternal distention and swelling of the mitochondria, suggesting a somewhat different mechanism than that of BPA (Figure 2, Tm; [56]).

Growing evidence from several experimental setups has shown that BPA may alter lipid regulation, mainly by interfering with insulin-mediated pathways [57]. Also, BPA, even at low, environmentally relevant doses, promotes lipid accumulation [58]. Thus, in BPA-treated cells, the occurrence of the autophagy intermediates bearing lipid-like droplets and the presence of cholesterol crystal clefts (spaces where cholesterol crystals have been dissolved during sample preparation) might be related to the detrimental effect of BPA on lipid regulation and lipid metabolism (Figure 2).

**Figure 8:**
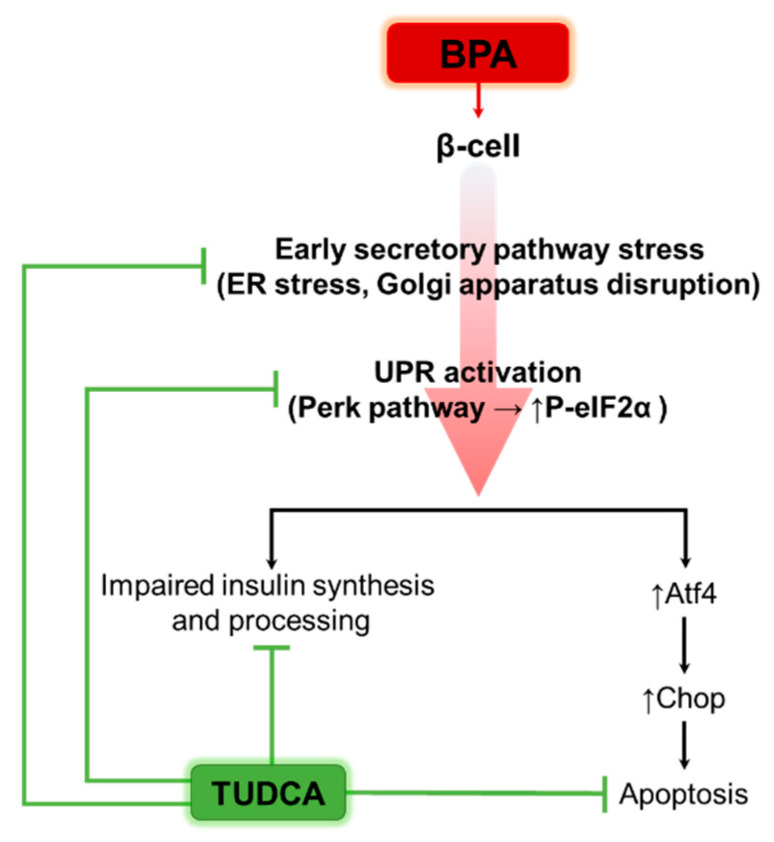
Schematic representation of the proposed actions of BPA and TUDCA on the β-cells.

In our data, *Ins* mRNA expression coupled with proinsulin and insulin immunofluorescence staining showed a significant decrease induced by higher doses of BPA on the whole insulin synthesis chain, suggesting a regulation that occurs at the transcriptional level (Figure 3). On the other hand, low dose BPA (10 µM) slightly increased the amount of *Ins2* mRNA both in MIN6 cells and in isolated mouse islets (Figure 3B,C and Figure 8). These data are in line with results obtained in other models, INS-1 cells or isolated rat islets, where researchers found that *Ins2* mRNA was increased at low concentrations of BPA while at higher concentrations was heavily reduced. This variation was confirmed also for insulin protein levels [18,48].

We proposed that one pathway responsible for the *Ins2* mRNA reduction could be the induction of the UPR that occurs in the cells exposed to BPA. Indeed, MIN6 cells treated with BPA show a predominant upregulation of the Perk UPR pathway, as opposed to Atf6 or Ire1 UPR branches. After 8 h of BPA treatment, we found high P-eIF2α, which is responsible for reducing global translation to relieve the ER de novo protein synthesis burden, including here *Ins2* mRNA, as was also shown before [59,60].

Our results showed that the UPR activation in response to BPA is very dynamic. The downstream targets, Atf4 and Chop, were induced in a time-dependent fashion by the treatment with BPA; however, we found their induction was somewhat not correlated (Figure 4). Atf4, the transcription factor found immediately downstream of P-eIF2α, steadily increased in time (we followed cells treated with BPA for up to 48 h) (Figure 4A and Figure S5). On the other hand, *Ddit3* (the gene coding for Chop) displayed a peak in expression at 12 h of BPA exposure, also found significantly at the corresponding Chop protein level (at 8 h), and by 48 h it returned to the 2 h values (Figure 4). Interestingly, after 24 h of BPA treatment, although the ratio P-Perk/Perk was increased, P-eIF2α/eIF2α was lower; however, Chop was nearly undetected.

Another possible BPA-induced mechanism of apoptosis could be related to the simultaneous upregulation of Atf4 and Chop (Figure 4A,B and Figure S5) and downregulation of Bip at the protein level but not at the mRNA level, supporting the P-Perk/P-eIF2α axis involvement (Figure 4B,C and Figure S3). This is a clear indication that MIN6 cells after 8 h of BPA exposure displayed a reduced adaptive UPR, and instead, a pro-apoptotic UPR was induced. Atf4 can influence the transcription of pro-apoptotic proteins from the Bcl-2 family, such as Bax, while downregulating the anti-apoptotic Bcl-2 [61], as we also observed from our experiments (Figure S6). Meanwhile, a downregulation of Bip can accelerate the apoptosis events. Besides its chaperon activity, Bip is a protein that has a role in maintaining cell viability by forming a complex with caspase-7, suppressing the pro-apoptotic events [62]. Surprisingly, we found that the dramatic downregulation of Bip observed at 8 h of BPA exposure is rescued by 24 h (Figure 4B,C), similar to the results obtained by Makaji and collaborators [50], which could indicate an adaptive mechanism.

TUDCA was previously employed by other researchers to restore insulin homeostasis through improving the adaptive UPR response of the β-cell in various diabetes models [36,37,63]. Our results showed a significant improvement in the viability of the MIN6 cells when TUDCA was added to the BPA treatment (Figure 5). Moreover, we found a decrease in P-eIF2α/eIF2α by 8 h of BPA exposure, albeit not significant, but might be sufficient to be physiologically relevant (Figure 6). This decrease was maintained by 24 h of BPA + TUDCA treatment, as we found both lower P-Perk/Perk and P-eIF2α/eIF2α as compared to BPA treatment alone. Surprisingly, both Atf4 and Chop were significantly even more upregulated by the co-exposure of MIN6 cells to both BPA and TUDCA than to BPA alone (Figure 6 and Figure S5). Moreover, we found that the upregulation of these transcription factors did not have an effect on the downstream targets, as neither *Bcl-2* (with a protector role against apoptosis) nor *Bax* and *Bad* (pro-apoptotic factors) varied significantly (Figure S5). Similar to our results, in GRP78/Bip conditional knockout mice’s Purkinje cells, Chop was induced but interestingly, the P-eIF2α levels were decreased, which was attributed to an upregulation in the levels of GADD34, an inhibitor of eIF2α phosphorylation [64]. Thus, it could be that there are other pathways responsible for the anti-apoptotic effect that TUDCA has in MIN6 cells.

Lastly, based on our results, we obtained an indication of another protective mechanism that TUDCA exerts in MIN6 cells. Upon evaluation of insulin synthesis and processing, we found that the addition of TUDCA in BPA-treated cells significantly upregulated both proinsulin and insulin expression as compared to BPA alone, suggesting an improved insulin processing (Figure 7A–C and Figure 8). These results were validated by electron microscopy, where MIN6 cells co-treated with BPA and TUDCA displayed a significantly increased number of mature insulin granules and a regular rough ER in comparison with cells exposed to BPA-only (Figure 7D,E and Figure 8). These are important findings because the data on ultrastructural defects caused by BPA in β-cells is rather scarce. To these, we also add our findings regarding the beneficial effects of TUDCA treatment on the insulin early secretory pathway of MIN6 cells.

Summing up all our data, we could propose TUDCA as a potent agent for enhancing β-cell survival, morphology and function by modulating UPR in a context characteristic to stressful environments, such as the one produced by the endocrine disruptor BPA (Figure 8).

## 4. Materials and Methods

### 4.1. Chemicals

We used the following chemicals: Bisphenol A (BPA, #239658), bovine serum albumin (BSA, #A7030) from Sigma-Aldrich (St. Louis, MO, USA); Tunicamycin (Tm, #654380) and tauroursodeoxycholic acid (TUDCA, #580549) from EMD Millipore, Sigma-Aldrich; Annexin V-APC (#640941) from BioLegend (San Diego, CA, USA) and propidium iodide (PI, #BMS500PI) from ThermoFisher Scientific (Waltham, MA, USA). All the other chemicals were purchased from Carl Roth (Karlsruhe, Germany). Dimethyl sulfoxide (DMSO, #A994.2) was used as solvent for Tm.

### 4.2. Antibodies

The following antibodies were used for immunofluorescence: mouse monoclonal anti-human/mouse proinsulin biotinylated antibody (1:200; R&D Systems (Minneapolis, MN, USA), #BAM13361), mouse monoclonal anti-calnexin antibody (1:100; Novus Biologicals (Englewood, CO, USA), #NB300-518), rabbit polyclonal anti-GM130 antibody (1:200; Novus Biologicals, #NBP2-53420), guinea pig polyclonal anti-insulin antibody (1:50; GeneTex (Zeeland, MI, USA), #GTX27842). Secondary antibodies were from ThermoFisher Scientific: anti-streptavidin AlexaFluor-568 (1:200; #S11226), AlexaFluor-488 (1:200; #A11001 and #A11073) and AlexaFluor-568 (1:200; #A10042).

The antibodies used for immunoblotting were the following: mouse monoclonal anti-actin antibody (C4) (1:500), rat monoclonal anti-GRP78/Bip (1:250) and mouse monoclonal anti-vinculin (1:1000) from Santa Cruz Biotechnology (Dallas, TX, USA), rabbit polyclonal anti-Phospho-eIF2*α* (1:500), rabbit polyclonal anti-eIF2*α* (1:500) and mouse monoclonal anti-Chop (clone L63F7) (1:200) from Cell Signaling (Danvers, MA, USA), rabbit polyclonal anti-Atf4 (1:1000) from GeneTex. Secondary antibodies conjugated with HRP were from Biolegend (anti-mouse and anti-rabbit) and from Santa Cruz Biotechnology (anti-rat). Standard blocking conditions (5% milk in TBS-T) were used throughout, except when anti-P-eIF2*α* antibody was used, and 1% BSA in TBS-T was utilized.

### 4.3. Cell Culture, Drug Treatment and Transfection

MIN6 cells were a kind gift from Pedro Herrera’s lab and used at passages between 21–29. Cells were cultured in DMEM high glucose (PAN-Biotech, Aidenbach, Germany), supplemented with 15% FBS (PAN-Biotech), 1% penicillin/streptomycin (PAN-Biotech) and 71 μM β-mercaptoethanol (culture medium). Media was changed every 2–3 days and cells were passaged once per week. For treatments, cells were passaged and left to adhere overnight. The next day, the medium was replaced with a culture medium containing different BPA concentrations (10, 50, 100, 250, 500 µM), 5 µg/mL Tm, 250 µM TUDCA, 100 µM BPA + 250 µM TUDCA, 250 µM BPA + 250 µM TUDCA. The vehicles used to dissolve the drugs were the following: 96% ethanol for BPA, distilled water for TUDCA and DMSO for Tm, respectively, and it never exceeded 0.5% from the culture medium. Treatments were maintained for 24 h or otherwise as stated in the results and figures sections. MIN6 cells were transfected 24 h after passage. Plasmid pLVX-ATF4 mScarlet NLS was a gift from David Andrews (Addgene plasmid # 115969; http://n2t.net/addgene:115969, accessed on 2 May 2022; RRID: Addgene_115969) [65] and was transfected using Lipofectamine 2000 (ThermoFisher Scientific) following manufacturer’s instructions. After 24 h incubation, transfection media was replaced with culture media containing 10–250 μM BPA or 5 μg/mL Tm for 6 h, before imaging using an Olympus CKX41 inverted microscope (Shinjuku City, Tokyo, Japan) with an Olympus XC30 camera.

### 4.4. Mice

Male NOR (non-obese diabetic resistant) mice were obtained from The Jackson Laboratory (Bar Harbor, ME, USA) (NOR/LtJ, stock #002050) and housed under specific pathogen-free conditions in our Animal Facility. Mice were kept under controlled temperature (21 °C), humidity (55–60%) and light conditions (12 h light:12 h darkness cycle) and with *ad libitum* access to food and water. All experimental procedures involving animals were conducted in accordance with the European Union Directive 2010/63/EU and approved by the national competent authority (Authorization No. 590/13.01.2021).

### 4.5. Islet Isolation and Culture

Islets were isolated from NOR mice. Briefly, the pancreas was injected through the papilla of Vaters with 2.5 mL Collagenase XI solution: Collagenase XI (Sigma-Aldrich, #C7657) dissolved in HBSS buffer supplemented with Ca^2+^, Mg^2+^ (CarlRoth, #9119.1) and 0.08% BSA. The harvested pancreas was incubated in a water bath at 37 °C for 15 min. The digestion was stopped by the addition of cold RPMI medium (Corning (Corning, NY, USA), #10–040-CV) supplemented with 10% FBS. Following digestion, the pancreas was transferred through a metallic strainer and washed two times with RPMI. Islet separation was performed with 10 mL of Histopaque-1077 (Sigma-Aldrich, #10771) overlaid with 5 mL RPMI and centrifuged at 850× *g* for 15 min. Islets are transferred from the gradient with a pipette to a new tube with RPMI. Islets were washed three times with RPMI, resuspended in RPMI with 10% FBS and hand-picked. For treatments, islets were cultured in DMEM low glucose (PAN-Biotech), supplemented with 10% FBS and 1% penicillin/streptomycin, for the indicated time.

### 4.6. Apoptosis and Death Assays

MIN6 cells were cultured in 6-well plates at a density of 3 × 10^5^ cells/well. After 24 h treatment with various concentrations of BPA (10–500 µM), 5 µg/mL Tm, 250 µM TUDCA, 100 µM BPA + 250 µM TUDCA, 250 µM BPA + 250 µM TUDCA, respectively, the culture medium containing the floating and dead cells was collected and the remaining cells were detached with 0.125% Trypsin (PAN-Biotech) and pooled with the collected medium. After centrifugation, cells were double stained with Annexin V-APC and PI, following manufacturer’s instructions. Cells were evaluated by flow cytometry with CytoFLEX (Beckman Coulter, Indianapolis, IN, USA). In total, 50,000 events were counted for every sample. The analysis consisted in gating out the debris, followed by evaluation of the percentages of cell populations equivalent for each group as follows: dead (PI positive), late apoptotic (PI and Annexin V positive), early apoptotic (Annexin V positive) and viable cells (negative to PI and Annexin V). Islets from NOR mice were cultured in 6-well plates (50 islets/plate) with BPA (10–250 µM) and 5 µg/mL Tm overnight. Islets were stained with PI and the dye intensity was quantified using Fiji/ImageJ software 1.51J8.

### 4.7. Microscopy and Image Analysis

Cells were cultured on glass coverslips in 6-well plates. After 24 h of exposure to various reagents (as reported in the figure legends), cells were washed with PBS, fixed with 4% PFA in PBS and processed for immunofluorescence. Briefly, cells were washed with PBS, treated with 50 mM NH_4_Cl for 10 min and permeabilized with 0.5% Triton X-100 for 20 min. In total, 1% BSA in PBS was used as a blocking buffer for 30 min, while the endogenous biotin was blocked with a biotin/avidin kit (BioLegend, #SIG-31126) for the cells stained with anti-proinsulin antibody. Cells were incubated with primary antibodies for 1 h at room temperature, washed 3 times with PBS and stained with secondary antibodies for 45 min at room temperature, followed by 3 washes with PBS. Cells were stained with DAPI (ThermoFisher Scientific) for 5 min, washed with PBS and rinsed with distilled water. Vectashield (Vector Laboratories (Burlingame, CA, USA), H-1000) was used as a mounting medium. Images of cells labeled for proinsulin and insulin were obtained on a fluorescence microscope Leica DMi8 (Leica Microsystems, Wetzlar, Germany) and were minimally processed using LasX software (version 3.7.6.25997). Fluorescence quantification was performed using QuPath software (version 0.3.2) [66] on images acquired in the same imaging session. The mean intensity of each fluorescence channel (red for proinsulin and green for insulin, respectively) was measured for each individual cell that was identified based on the nuclei staining with DAPI (blue). Brightfield images of cells and of mouse pancreatic islets, as well as cells stained with PI, or transfected with pLVX-ATF4 mScarlet NLS, were obtained by using an Olympus CKX41 inverted microscope with an Olympus XC30 camera. Similarly, images used for quantification were taken in the same imaging session and were processed and quantified by Fiji/ImageJ. The graphs were generated with GraphPad Prism (version 9) (GraphPad Software, San Diego, CA, USA).

### 4.8. Reverse Transcription and qPCR

For total RNA isolation, cells and islets were collected in 1 mL TRItidy G (PanReac AppliChem (Chicago, IL, USA), #A4051) and stored at −80 °C. Samples were mixed with 0.2 mL chloroform, kept on ice for 10 min, and then centrifuged for 15 min at 12,000× *g* at 4 °C. The aqueous phase was transferred to a new tube and incubated with isopropanol for 30 min on ice. Samples were centrifuged for 15 min, at 12,000× *g* in a centrifuge kept at 4 °C. The RNA pellet was washed with 70% ethanol and with 100% ethanol, respectively. After air drying, the pellet was resuspended in 20 μL DEPC-water. RNA purity and concentration were evaluated by NanoDrop (ThermoFisher Scientific). For reverse-transcription reaction, 1 μg RNA was used and the cDNA was obtained with 9902 Veriti PCR (Applied Biosystems, Waltham, MA, USA) by using a qScript cDNA SuperMix (Quantabio, Beverly, MA, USA) kit or iScript Reverse Transcription Supermix (BioRad, Hercules, CA, USA). To evaluate gene expression, qPCR was performed, where the equivalent of 12.5 ng of cDNA was used with a Roche LightCycler 480 System (Roche, Basel, Switzerland) and PerfeCTa SYBR Green SuperMix kit (Quantabio). The primer sequences for the genes analyzed are found in Table 1.

### 4.9. Western Blot

Cells cultured in 6-well plates were lysed using RIPA buffer (150 mM NaCl, 50 mM TRIS, 1% Nonidet P-40, 1% Sodium deoxycholate, 0.1% SDS, 1 mM EDTA, 10% glycerol) supplemented with proteases inhibitors (1 μg/mL leupeptin, 1 μg/mL pepstatin, 1 μg/mL benzamidine) and phosphatases inhibitors (5 mM NaF, 5 mM beta-glycerophosphate, 1 mM Na3VO4). Total protein was quantified using the ROTI Quant Universal kit. After normalization, samples were mixed with Laemmli 5× (S × 5) supplemented with 5% β-mercaptoethanol, boiled for 5 min at 95 °C and loaded in 10% SDS-PAGE gels. Proteins were transferred on a PVDF membrane using a Trans-Blot Turbo Transfer System (BioRad). Membranes were blocked with 5% skimmed milk in TBS-Tween 0.05% (TBST) or 1% BSA in TBST for 1 h at room temperature. Membranes were incubated with the antibodies presented above, then with ECL solution (1 M Tris-HCl pH 8.5, 250 mM luminol, 90 mM coumaric acid, and 30% hydrogen peroxide) and visualized using LAS-4000 FujiFilm (GE Healthcare, Chicago, IL, USA).

### 4.10. Transmission Electron Microscopy Evaluation

For ultrastructural analysis, MIN6 cells treated as described were fixed with 2.5% glutaraldehyde in 0.1 M sodium cacodylate buffer, post-fixed in 1% osmium tetroxide and stained “en bloc” with 1% uranyl acetate. Cells were dehydrated through a graded series of ethanol, transferred to propylene oxide and embedded in Epon 812. Ultrathin 70 nm sections were cut with an ultramicrotome, placed on formvar-coated copper grids (200 mesh) and double-stained with uranyl acetate and lead citrate before examination on an electron microscope (Tecnai G2 Spirit BioTwin, ThermoFisher Scientific, Eindhoven, The Netherlands) at 100 kV. Images were acquired with an Eagle 4K bottom-mounted CCD camera and analyzed using Fiji/ImageJ (v1.53) software [77]. For each experimental condition, at least 10 images taken at lower magnification (2900× to 6800×) were considered for quantitative evaluation of the insulin secretory granules. Granule density was defined as the granule number per micron squared of cytosol. Cytosol and dense-core area were quantified using the Set Measurements function in Fiji/ImageJ. Graphs were generated with GraphPad Prism.

### 4.11. Statistical Analysis

The results were quantified and the mean or median of 3 or more biological replicates was evaluated. Statistical analysis was performed using GraphPad Prism software. One-way ANOVA was performed and the differences between groups were obtained with the Tukey test. A significant difference was considered to be for *p* < 0.05 (differences *p* < 0.05 are denoted by *, *p* < 0.01 are denoted by **, *p* < 0.001 are denoted by ***, *p* < 0.0001 are denoted by ****). The results are shown as means ± SD, exceptions are graphs in Figure 3E,F, Figure 6B,C and Figure S5 where the median and the 25th and 75th percentiles are presented.

## Figures and Tables

**Table 1:** Primers sequences.

Gene	Forward Primer 5′-3′	Reverse Primer 3′-5′	Reference
*Gapdh*	TCCATGACAACTTTGGCATTG	CAGTCTTCTGGGTGGCAGTGA	[67]
*Atf4*	ATGGCCGGCTATGGATGAT	CGAAGTCAAACTCTTTCAGATCCATT	[68]
*Ddit3*	CACATCCCAAAGCCCTCG	CTCAGTCCCCTCCTCAGC	[69]
*Bcl-2*	TCGCAGAGATGTCCAGTCAG	ATGCCGGTTCAGGTACTCAG	[70]
*Bax*	CAGGATGCGTCCACCAAGAA	CGTGTCCACGTCAGCAATCA	[71]
*Bad*	GCCCTAGGCTTGAGGAAGTC	CAAACTCTGGGATCTGGAACA	[71]
*Bip*	AGGACAAGAAGGAGGATGTGGG	ACCGAAGGGTCATTCCAAGTG	[72]
*uXbp1*	CAGCACTCAGACTATGTGCA	GTCCATGGGAAGATGTTCTGG	[73]
*sXbp1*	CTGAGTCCGCAGCAGGTGCAG	GTCCATGGGAAGATGTTCTGG	[73]
*Atf6*	GACTCACCCATCCGAGTTGTG	CTCCCAGTCTTCATCTGGTCC	[74]
*Edem1*	CTGCAATGAAGGAGAAGGAG	TAGAAGGCGTGTAGGCAGAT	[75]
*Dnajc3*	TCCTGGTGGACCTGCAGTACG	CTGCGAGTAATTTCTTCCCC	[76]
*Ins2*	TCAACATGGCCCTGTGGAT	AAAGGTGCTGCTTGACAAAAGC	[67]

## Data Availability

Data are contained and available within this manuscript.

## Supplementary Materials

The following supporting information can be downloaded at: https://www.mdpi.com/article/10.3390/ijms24032023/s1.
