# Supplementary material for: Modulation of Unfolded Protein Response Restores Survival and Function of β-Cells Exposed to the Endocrine Disruptor Bisphenol A
Source: Int J Mol Sci. 2023 Jan 19;24(3):2023. doi: 10.3390/ijms24032023 (PMC9916570; doi:10.3390/ijms24032023)
Supplement: Supplementary file 1 [file ijms-24-02023-s001.zip › ijms-2164994-supplementary.pdf]

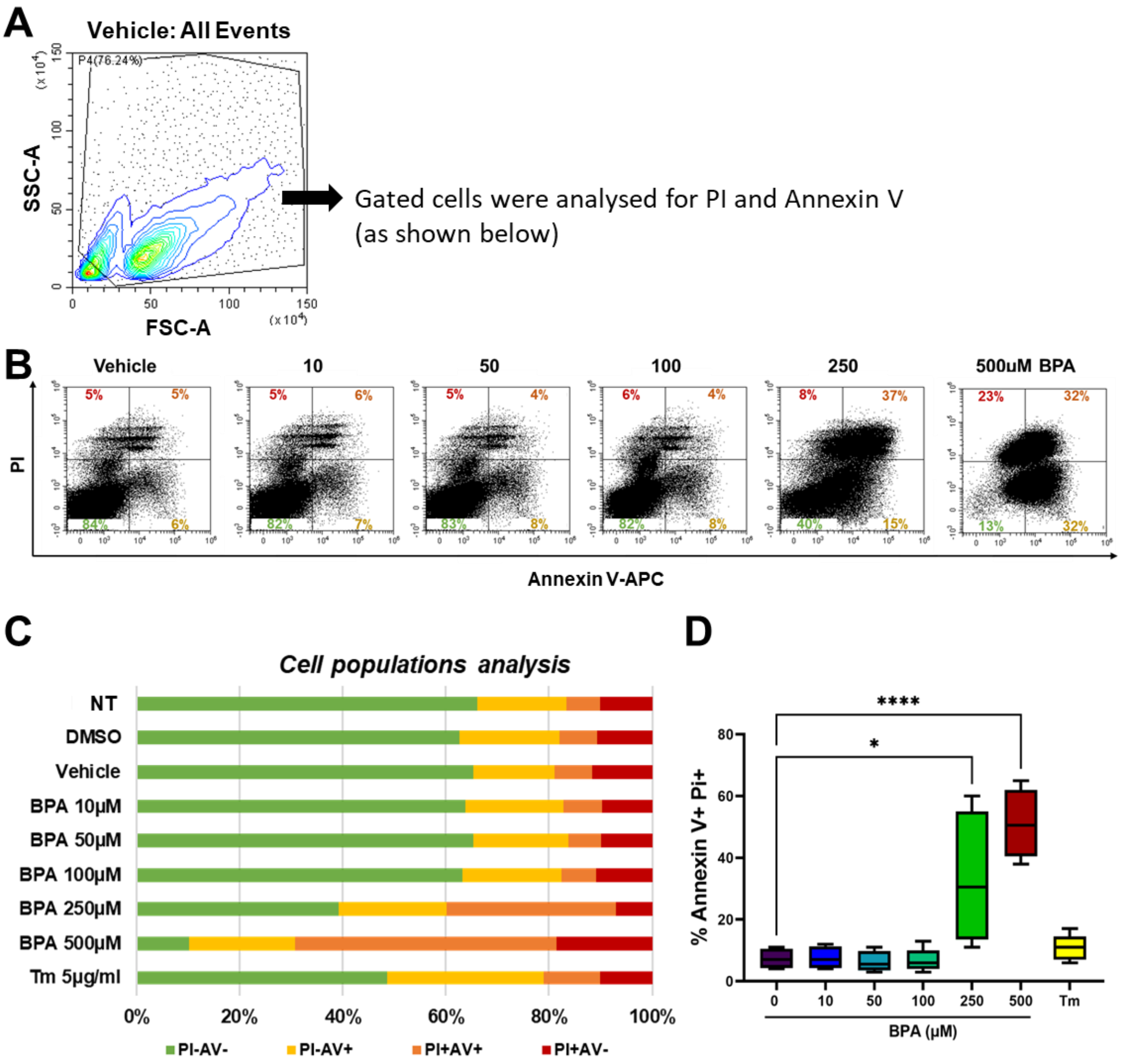

Figure S1. Cell viability was decreased after 24h treatment with BPA and Tm. (**A**) Gating strategy used in the flow cytometry analysis for the whole population of MIN6 cells treated with increasing concentrations of BPA (10-500 µM) and Tm (5 µg/ml). (**B**) Representative flow cytometry dot-plots depicting cells stained with PI (y axis) and Annexin V (x axis). Percentages of each labelled population are marked in the corresponding quadrant and they can be found correlated in the graph in (**C**) Graph depicting apoptotic and dead cells determined by flow cytometry analysis upon staining with Annexin V and PI for MIN6 cells treated with BPA (0-500 µM) or with 5 µg/ml Tm (**D**).

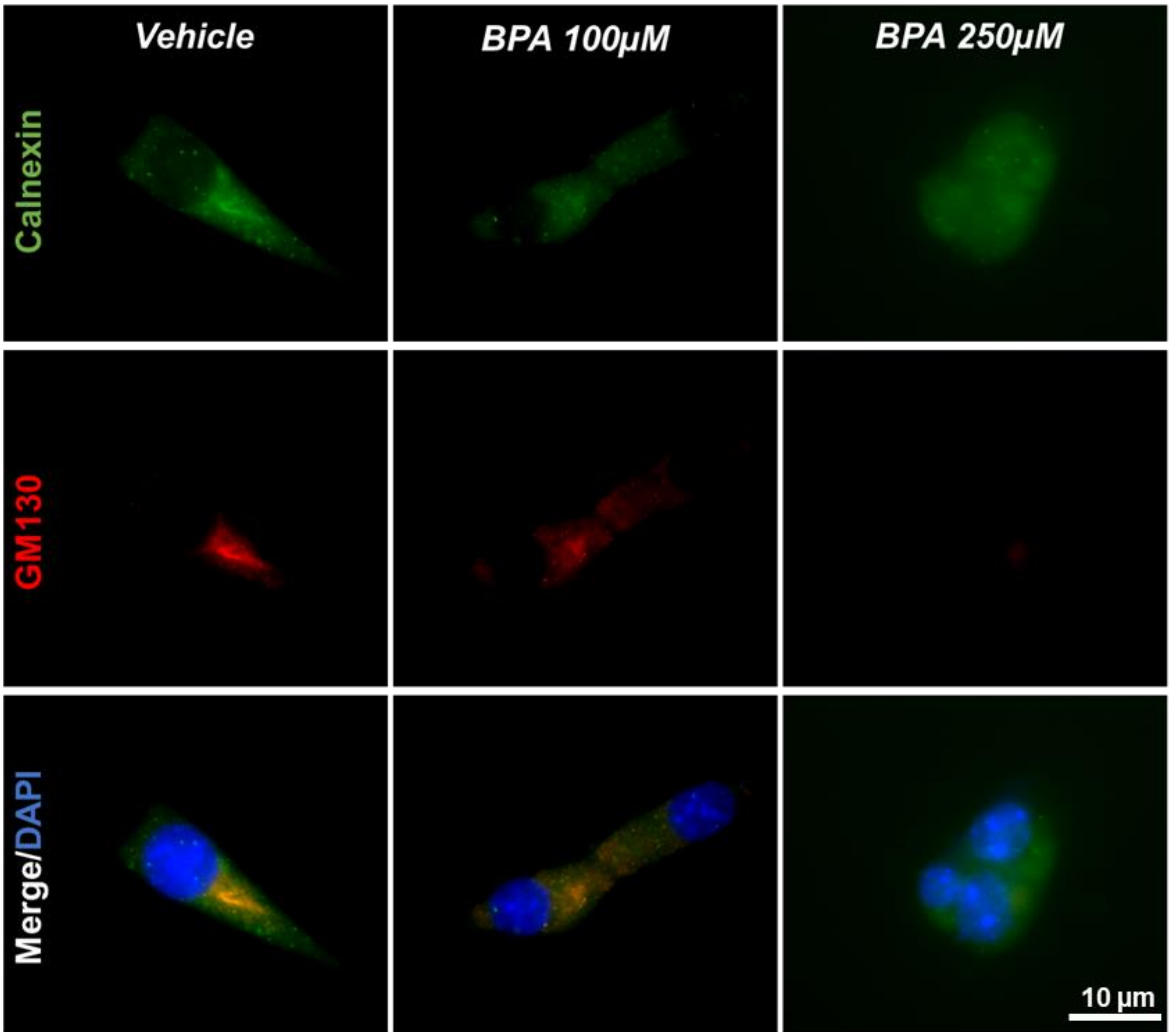

Figure S2. **BPA determines the disruption of the early secretory pathway in MIN6 cells.** Fluorescence images of MIN6 cells after 24 h of treatment with 100 and 250 μM BPA, respectively, were immunostained for calnexin (green), an ER marker, and for GM130 (red), a cis-Golgi apparatus marker. The nuclei were stained with DAPI (blue). Scale bar is 10 μm.

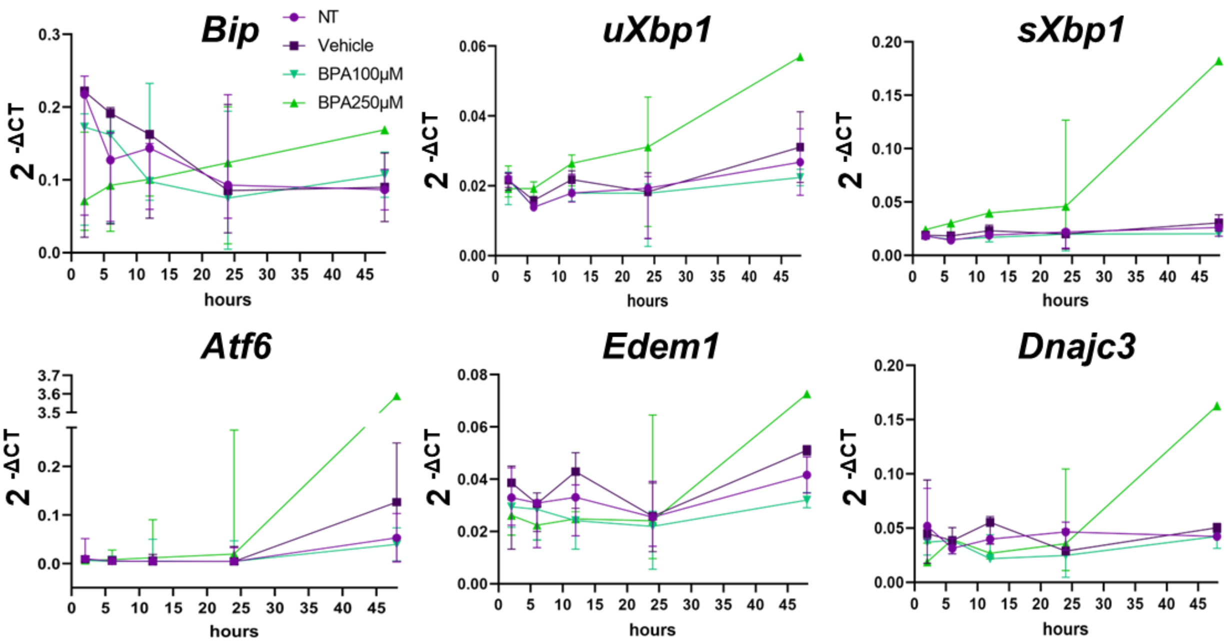

Figure S3. Ire1 and Atf6 pathways were not induced after 24 h of BPA treatment in MIN6 cells. Time course of BPA-induced gene expression of *Bip, uXbp1, sXbp1, Atf6, Edem1* and *Dnajc3* in MIN6 cells. $2^{-\Delta CT}$ values were calculated by normalization to *Gapdh*.

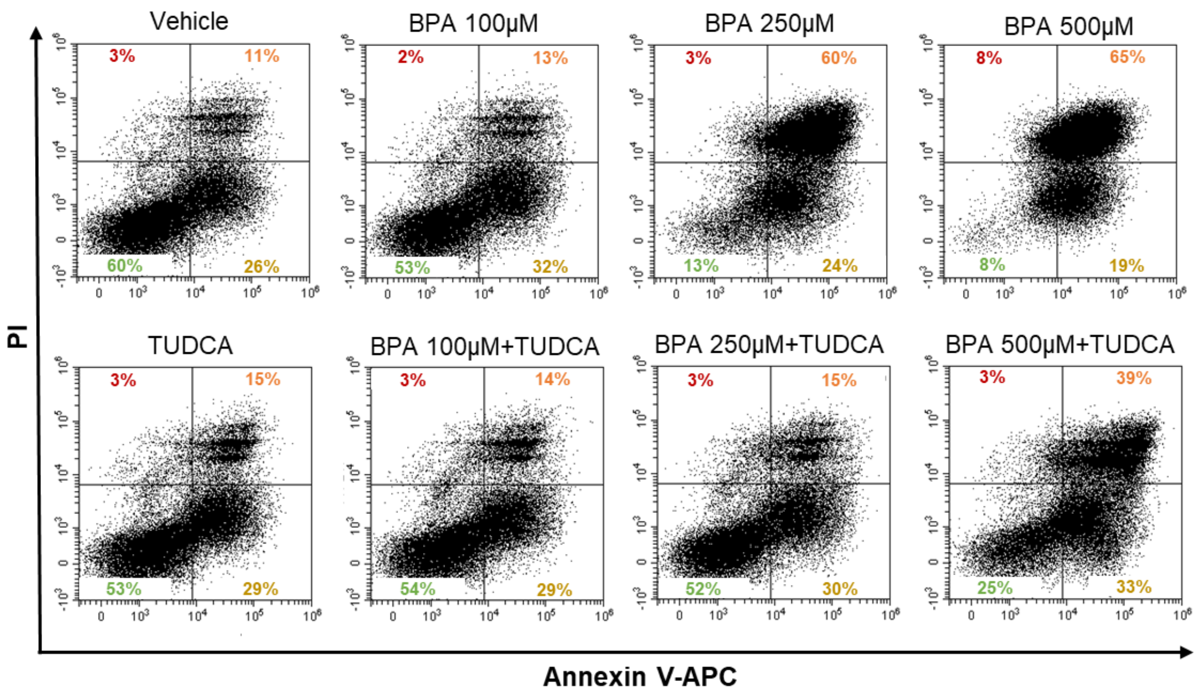

Figure S4. TUDCA rescues MIN6 cells viability after 24h co-treatment with BPA. Representative flow cytometry dot-plots depicting cells stained with PI (y axis) and Annexin V (x axis) and treated as shown.

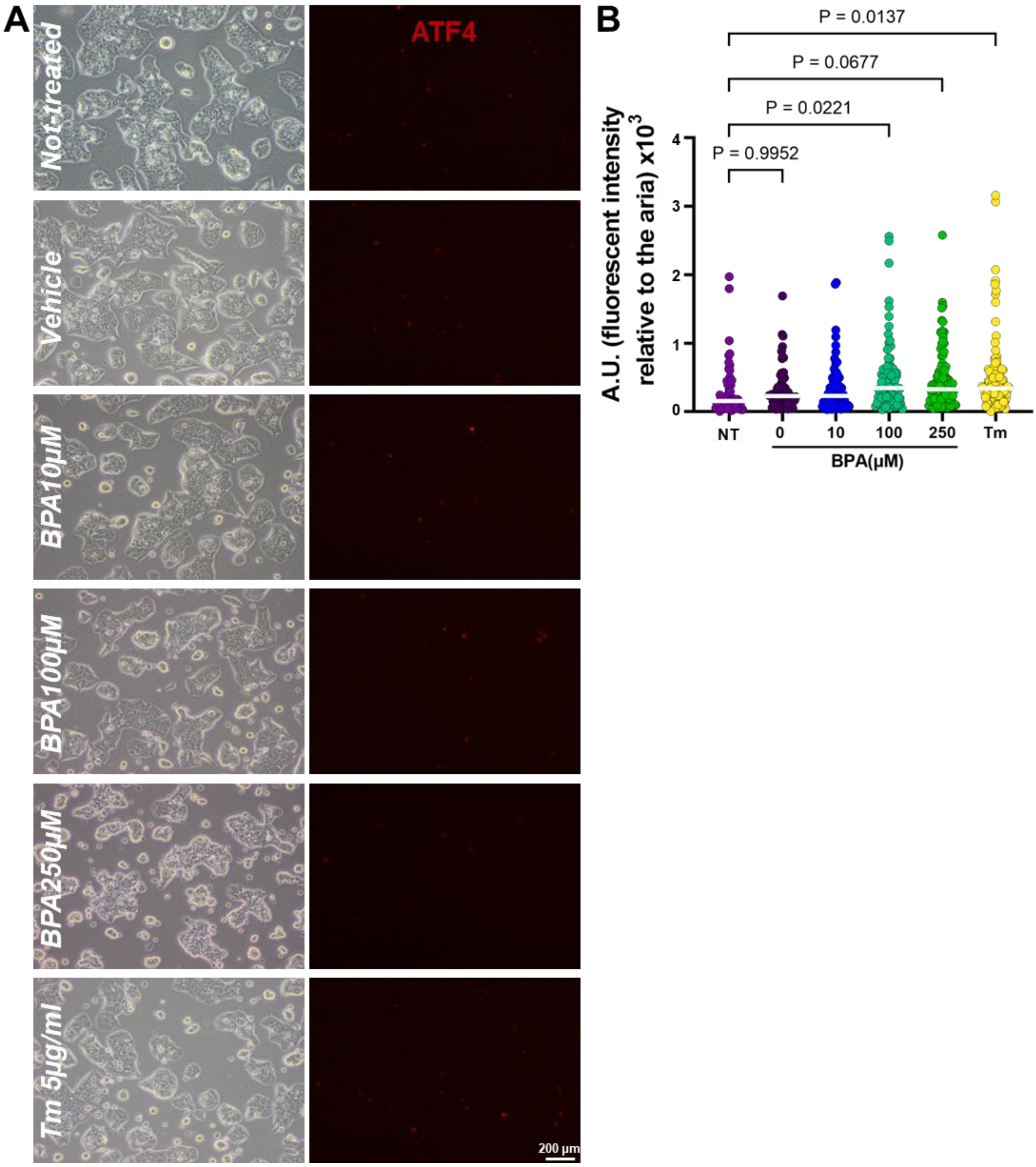

Figure S5. ATF4 activation in MIN6 cells (**A**) Representative brightfield and fluorescent microscopy images of MIN6 cells transfected with pLVX-ATF4 mScarlet NLS plasmid, after 6 h of treatment with 10-250 µM BPA and 5 µg/ml Tm. (**B**) Graphic representation of the quantification of mean fluorescence intensity relative to the area of the cell clusters. Graph displays the median and the 25th and 75th percentiles. Statistical analysis by One-way ANOVA.

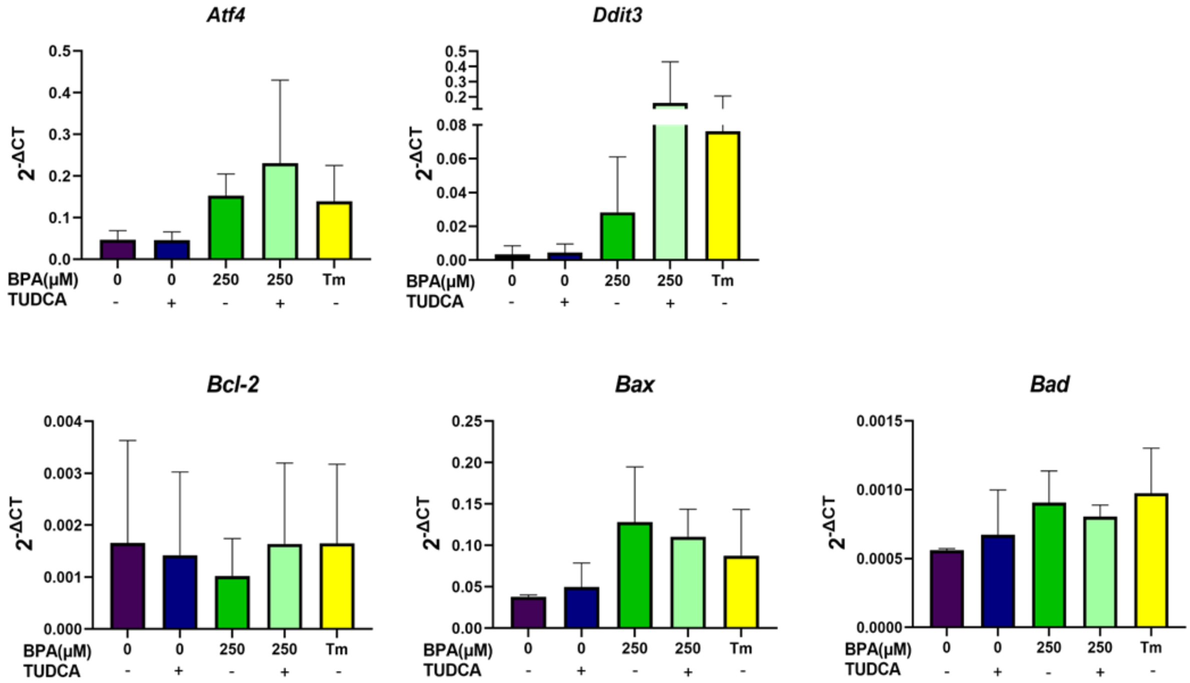

Figure S6. The pro-apoptotic effectors gene expression variation following co-treatment with BPA and TUDCA of MIN6 cells. RT-qPCR analysis of *Atf4, Ddit3 Bcl-2*, *Bax* and *Bad* genes in MIN6 cells after 24 h of co-treatment with BPA and TUDCA and 5 µg/ml Tm; $2^{-\Delta CT}$ values and were calculated by normalization to *Gapdh.*
